# TPI1 activates the PI3K/AKT/mTOR signaling pathway to induce breast cancer progression by stabilizing CDCA5

**DOI:** 10.1186/s12967-022-03370-2

**Published:** 2022-05-04

**Authors:** Xiaoying Jin, Dandan Wang, Mengxia Lei, Yan Guo, Yuqing Cui, Fengzhi Chen, Weiling Sun, Xuesong Chen

**Affiliations:** 1 The Fourth Department of Medical Oncology, Harbin Medical University Cancer Hospital, Harbin, 150040 China; 2 Department of Medical Oncology, Harbin Medical University Cancer Hospital, Harbin, 150040 China

**Keywords:** TPI1, CDCA5, Glycolysis, Epithelial–mesenchymal transformation (EMT), PI3K/AKT/mTOR

## Abstract

**Background:**

Triosephosphate isomerase 1 (TPI1), as a key glycolytic enzyme, is upregulated in multiple cancers. However, expression profile and regulatory mechanism of TPI1 in breast cancer (BRCA) remain mysterious.

**Methods:**

Western blotting and immunohistochemistry (IHC) assays were used to investigate the expression of TPI1 in BRCA specimens and cell lines. TPI1 correlation with the clinicopathological characteristics and prognosis of 362 BRCA patients was analyzed using a tissue microarray. Overexpression and knockdown function experiments in cells and mice models were performed to elucidate the function and mechanisms of TPI1-induced BRCA progression. Related molecular mechanisms were clarified using co-IP, IF, mass spectrometric analysis, and ubiquitination assay.

**Results:**

We have found TPI1 is highly expressed in BRCA tissue and cell lines, acting as an independent indicator for prognosis in BRCA patients. TPI1 promotes BRCA cell glycolysis, proliferation and metastasis in vitro and in vivo. Mechanistically, TPI1 activates phosphoinositide 3-kinase (PI3K)/AKT/mammalian target of rapamycin (mTOR) pathway to regulate epithelial–mesenchymal transformation (EMT) and aerobic glycolysis, which is positively mediated by cell division cycle associated 5 (CDCA5). Moreover, TPI1 interacts with sequestosome-1 (SQSTM1)/P62, and P62 decreases the protein expression of TPI1 by promoting its ubiquitination in MDA-MB-231 cells.

**Conclusions:**

TPI1 promotes BRCA progression by stabilizing CDCA5, which then activates the PI3K/AKT/mTOR pathway. P62 promotes ubiquitin-dependent proteasome degradation of TPI1. Collectively, TPI1 promotes tumor development and progression, which may serve as a therapeutic target for BRCA.

**Supplementary Information:**

The online version contains supplementary material available at 10.1186/s12967-022-03370-2.

## Introduction

Global incidence of breast cancer (BRCA) in women has increased significantly [1], which has surpassed lung cancer and become the most commonly diagnosed cancer, with an estimated 2.3 million new cases (accounting for 11.7% of all cancer cases) in 2020; BRCA remained as the leading cause of female cancer-related death, accounting for 6.9% of mortalities worldwide [2]. Despite advances in diagnosis and treatment, recurrence and death rates from BRCA remain high. Therefore, to improve overall survival and quality of life, it is urgent to discover new targets and to demonstrate molecular mechanisms for diagnosing and treating BRCA.

Energy metabolism in cancer has been extensively explored. Glycolysis, in particular, has become an attractive target for cancer therapy because many tumors exhibit a significant increase in glucose uptake compared with adjacent normal tissue [3]. 3-Bromopyruvate, as a glyceraldehyde-3-phosphate dehydrogenase (GAPDH) inhibitor, had exhibited anticancer efficacy in animal models [4, 5]. Glycolysis involves a series of enzymes [6], which may regulate downstream genes of mTOR signaling pathway. PKM2 activates mTORC1 signaling pathway by phosphorylating AKT1S1, which accelerates oncogenic growth in cancer cells [7, 8]. The phosphoinositide 3-kinase (PI3K)/ AKT serine/threonine kinase (AKT) signaling pathway is involved in many cellular processes, including metabolism, metastasis [9, 10] and epithelial–mesenchymal transformation (EMT) [11]. Therefore, targeting glycolytic multienzyme system and related pathways are hypothesized be a novel therapeutic strategy in BRCA.

Triosephosphate isomerase 1 (TPI1) is regarded as a key enzyme in the glucose metabolism pathway and is involved in various biological functions, especially in metastatic phenotype [12]. TPI1, also known as TIM, TPI, TPID, and HEL-S-49, is located in 12p13.31 [13, 14]. TPI1 encodes a triosephosphate isomerase consisting of two identical subunits, which catalyzes the interconversion between glyceraldehyde-3-phosphate and dihydroxy-acetone phosphate in glycolysis and gluconeogenesis pathway, respectively [15]. TPI1 has been proposed as a candidate oncogene, with its overexpression detected in several types of cancers, such as intrahepatic cholangiocarcinoma (ICC) [16], gastric [13], lung [17, 18], and prostate cancer [19]. In ICC, TPI1 was overexpressed and correlated with poor prognosis [16]. TPI1 was recognized as a serum biomarker in patients with cancer, including BRCA [20, 21], lung squamous cell carcinoma [17] and hepatocellular carcinoma (HCC) [22]. However, in HCC, TPI1 was identified as a tumor suppressor gene [23]. These controversial findings indicate that exact roles of TPI1 in cancers, in particular BRCA, are yet to be identified.

In this study, we found that TPI1 is a marker of poor prognosis in BRCA. By promoting cell division cycle associated 5 (CDCA5) protein stabilization, TPI1 activates the PI3K/AKT/mTOR pathway, thereby enhancing metastasis and glycolysis. In addition, we also discovered that the ubiquitin-associated protein P62, interacts with TPI1 and promotes ubiquitin-dependent proteasome degradation of TPI1 in MDA-MB-231cells.Our study not only demonstrates, for the first time, the role of TPI1 in promoting breast cancer progression, but also provides a new strategy for targeted glycolysis therapy for breast cancer.

## Materials and methods

### Bioinformatics analysis

In this study, The Cancer Genome Atlas Research Network (TCGA, http://cancergenome.nih.gov), UALCAN (http://ualcan.path.uab.edu) and METABRIC database were explored. We used the Kaplan–Meier plotter (KM plotter, http://kmplot.com) database and the survival R packages were employed to analyze clinical outcomes. Linkedomics [24], cBioportal (http://cbioportal.org) and Gene Expression Profiling Interactive Analysis (GEPIA, http://gepia2.cancer-pku.cn) databases were applied, alongside R4.0.5.

### Tissue specimens and patients

Totally 10 pairs of fresh breast tissues (paired BRCA tumor samples and matched adjacent normal tissue samples) were obtained from randomly selected patients for western blot (WB). We collected 28 normal samples and 362 BRCA samples from patients who were treated by surgical removal in 2007 for tissue microarray, with follow-up until July 2021.The overall survival (OS) and disease-free survival (DFS) were calculated as time from surgery until the occurrence of death and relapse, respectively. In addition, pathological sections from 50 BRCA patients were used for correlation analysis. All clinical samples were collected at Harbin Medical University Cancer Hospital and verified by histological and pathological examinations. This study was approved by the Ethics Committee of Harbin Medical University. All participating patients had provided written informed consent.

### Immunohistochemistry (IHC)

The IHC assay was performed as previously described [25]. Primary antibodies were listed in an Additional file 1: Table S1. The staining results were scored according to the following criteria: percentage of immunoreactive cells: 0 (0–5%), 1 (6–25%), 2 (26–50%), 3 (51–75%), or 4 (76–100%); and staining intensity: 0 (negative), 1 (weak), 2 (moderate), or 3 (intense). The final score for TPI1 expression was the multiplication of percentage and intensity. For statistical analysis, a final staining score of ≤ 7 was defined as low expression, whereas a score > 7 as high expression.

### Cell culture, transfection and treatment

Human BRCA cell line and MCF-10A were secured from the Cancer Research Institute of Heilongjiang Province. 10% fetal bovine serum (ExCell Bio, Australia) and 1% penicillin/streptomycin (Solarbio, China) were added to all media. MCF7, T47D, UACC-812, and HCC70 cells were cultured in DMEM (Gibco, Life Technologies, California, USA), whereas MDA-MB-453 and SKBR-3 cells in RPMI 1640 (Gibco, California, USA), and MCF-10A cells in complete medium purchased from Shanghai Zhongqiaoxinzhou Biotech (Shanghai, China [CAS: ZQ1311]). Cell lines were incubated in a humidified incubator at 37 °C with 5% CO_2_. MDA-MB-231 and MDA-MB-468 cells cultured in Leibovitz’s L-15 (PM151010, Procell, China) were placed in an incubator without CO_2_.

TPI1 knockdown (sh1, sh2, sh3) and vector (NC) by lentiviral system (Hanbio, Shanghai, China) with a puromycin selection marker were stably transfected into T47D cells. Furthermore, overexpressed TPI1 controlled with lentiviral system (Hanbio, Shanghai, China) was stably transfected into MDA-MB-231 cells according to the manufacturer’s instructions. Sequences of the interference TPI1 were listed in an Additional file 1: Table S2. Stable cells with lentiviral system were yielded after treatment with 1 μg/ml puromycin for 2 weeks. CDCA5 plasmid (CDCA5) and P62 plasmid (P62) were transfected into BRCA cells using jetPRIME® (Polyplus-transfection S.A, Illkirch, France) according to the manufacturer’s instructions, with empty vector plasmid as a negative control. All plasmids were purchased from Sino Biological (Beijing, China). Transfection efficiency was determined by qRT-PCR and WB, respectively. For cycloheximide (CHX) chase assay, cells were incubated with 200 μg/ml CHX (HY-12320, MCE, USA) for indicated durations (0, 2, 4, 6, 8, 10, and 12 h, respectively). LY294002 was used in cell-based assay (50 μM, 24 h) and mice models (75 mg/kg).

### Western blot (WB) and qRT-PCR

WB was conducted as previously described [25]. Briefly, cell lysates were obtained using RIPA lysis buffer (Beyotime, Shanghai, China). Concentrations of protein were confirmed by a BCA Protein Assay Kit (Thermo Scientific). Protein was separated using 10% or 12.5% SDS–polyacrylamide gel electrophoresis and transferred to polyvinylidene difluoride membranes. After blocking with 5% milk, the membranes were incubated with primary antibody overnight at 4 °C. The next day, it was incubated for 1 h at room temperature with corresponding secondary antibodies and then was visualizing by an ECL Plus kit (Xin Saimei, China). The primary antibodies were listed in Additional file 1: Table S3.

Total RNA was extracted using TRNzol reagent (TIANGEN Biotech, Beijing, China). Complementary DNA was synthesized using a high-capacity cDNA reverse transcription kit. qRT-PCR was conducted with the Applied Biosystems 7500 Real-Time PCR System using a SYBR Green Real-Time PCR Master Mix kit (Takara, Dalian, China). Primers for qRT-PCR were listed in Additional file 1: Table S4.

### Cell proliferation assays

For Cell Counting Kit-8 (CCK-8) assay, 5 × 10^3^ cells/well were seeded in 96-well plates (Jet Biofil, Guangzhou) and cultured at 37 °C. 10 μl of CCK-8 (SEVEN BIOTECH, China) was mixed with 90 μl of medium. The mixture was added to each well at time points of 1, 2, 3, 4, and 5 days, respectively, after seeding, incubated for 2 h, and measured for absorbance at 450 nm.

For colony formation assay, cells (8 × 10^2^/well) were seeded in 6-well plates, culture medium was changed every 2–3 days. Two weeks later, living cells were fixed, stained, and counted.

An Edu assay, a total of 5 × 10^3^ cells/each well were seeded in 96-well plates for 2 days, added with Edu at a concentration of 50 μM, and cultured 2 h at 37 °C before fixation. This assay was carried out following the manufacturer’s protocol of Click-IT Edu Imaging Kits (Yuheng, Suzhou, China). Images were taken under an inverted fluorescence microscope.

### Migration, invasion and wound healing assays

For migration assays, cells (3 × 10^4^–7 × 10^4^) were inserted in 200 μl of serum-free medium, and 600 μl medium with 10% FBS was added to the bottom chambers. After 24 h, cells were fixed in 4% paraformaldehyde and stained with crystal violet solution for 2 h. The number of cells per five randomly selected fields was counted under an inverted microscope (Leica, Germany). Then, cells were photographed and counted in five randomly selected fields.

For invasion assays, the upper basement membrane of the chamber was precoated with 30 μg Matrigel (356243, BD Biosciences) and cultured for 48 h at 37 °C. The procedure was the same as migration assay.

For wound healing assays, wounds were scratched on a monolayer cell by 10 μl pipette tips until 95% of the cells were covered in a 6-well plate. Then, wound healing images were taken at appropriate time points (24 h/48 h). The migration distance between the dotted lines was measured and normalized according to that of control cells.

### Glucose uptake and lactate production measurement

Cells were cultured in 96-well plates for 48 h to examine glucose consumption rate and lactate production. The supernatants were collected for detection, measured for absorbance at 490 nm, and calculated according to the manufacturer’s instructions. The Glucose Assay Kit (Nanjing Jiancheng Bioengineering Institute, Nanjing, China) and Lactate Assay Kit (A019-2-1, Nanjing Jiancheng Bioengineering Institute) were applied for respective experiments.

### In vivo assay

Female BALB/c nude mice (4–5 weeks old) were obtained from the Beijing Vital River Laboratory and housed under standard specific-pathogen-free conditions of the Animal Center of the Second Affiliated Hospital of Harbin Medical University.

The mice were randomly divided into three groups (n = 5/group). Two groups of mice were subcutaneously injected with MDA-MB-231 cells overexpressing TPI1-luciferase (5 × 10^6^ cells in 200 µl phosphate buffered saline (PBS)/Matrigel [3:1]), while the remaining group was injected with MDA-MB-231-luciferase vector control cells. After primary tumor formation, five mice from TPI1 overexpressing group were randomly selected to receive LY294002 (75 mg/kg) intragastric administration 3 times a week for 3 weeks. T47D vector cells or shTPI1 cells (5 × 10^6^ cells in 200 µl PBS/Matrigel [3:1]) were injected subcutaneously (n = 6/group). The tumor volume was monitored using vernier calipers every 4–5 days for a month, calculated by the formula (width^2^ × length)/2 (mm^3^). The mice were sacrificed after 35 days and tumor weight was measured. Tumor tissues were used for WB, H&E and IHC assays.

### Immunoprecipitation (IP), silver staining and mass spectrometry assay

The protein A/G magnetic beads (MCE, HY-K0202) were incubated with antibodies at 4 °C for 1 h, followed by incubation with cell lysates based on the instructions. Subsequently, the beads were collected and subjected to immunoprecipitation (IP). A silver staining assay was performed according to the protocol provided by Beyotime Technology (P0017S, Beyotime, Shanghai, China). The antibody, magnetic beads and antigen were combined and boiled at 95 °C, and then the mass spectrometry results were obtained by Wuhan GeneCreate Biological Engineering. The antibodies used for the co-IP were TPI1 (Proteintech, 10713-1-AP), CDCA5 (Santa Cruz, sc-365319), and sequestosome-1/P62 (SQSTM1/P62) (Proteintech, 66184-1-Ig).

### Immunofluorescence (IF) assays

The cells were fixed with 4% paraformaldehyde for 20 min, permeabilized with 0.3% Triton X-100 for 10 min, and blocked by 10% normal goat serum for 30 min at room temperature. Cells were washed 3 times with PBS. Primary antibody was administered overnight at 4 °C. The next day, the cells were incubated with secondary antibody for 1 h and stained with DAPI (Beyotime, Shanghai, China) for 5 min at room temperature in a dark room. During each step, cells were washed 3 times with PBST. Then, pictures were taken under a confocal microscope (ZEISS LSM 800). The antibodies used were CDCA5 (Proteintech, 1:80 dilution, 67418-1-Ig), TPI1 (Proteintech, 1:50 dilution, 10713-1-AP), DyLight 649 Goat Anti-Rabbit IgG (A23620, Abbkine) or DyLight 488, and Goat Anti-Mouse IgG (A23210, Abbkine).

### Ubiquitination assay

MDA-MB-231 cells transfected with Flag-Ub plasmid were pretreated with MG132 (10 μM) (MCE, New Jersey, USA) for 6 h and lysed. Then, co-IP was performed and analyzed by WB using an anti-ubiquitin antibody (1:100 dilution; ab134953, Abcam, Cambridge, MA, USA).

### Statistical analysis

We performed statistical analyses using SPSS 22.0 software and GraphPad Prism 8.0 software, collecting data from no fewer than three independent experiments. The statistical results were expressed as mean ± standard deviation. The differences between two groups were analyzed using the student’s t-test and the chi-square test. Survival was calculated using the KM plotter method and the log-rank test. Spearman’s rank correlation coefficient (r) was used to quantify correlation analysis. A *p* value of < 0.05 (two-tailed) was considered statistically significant.

## Results

### Glycolysis-associated gene TPI1 is overexpressed and correlates with a poor prognosis in BRCA

**Fig. 1:**
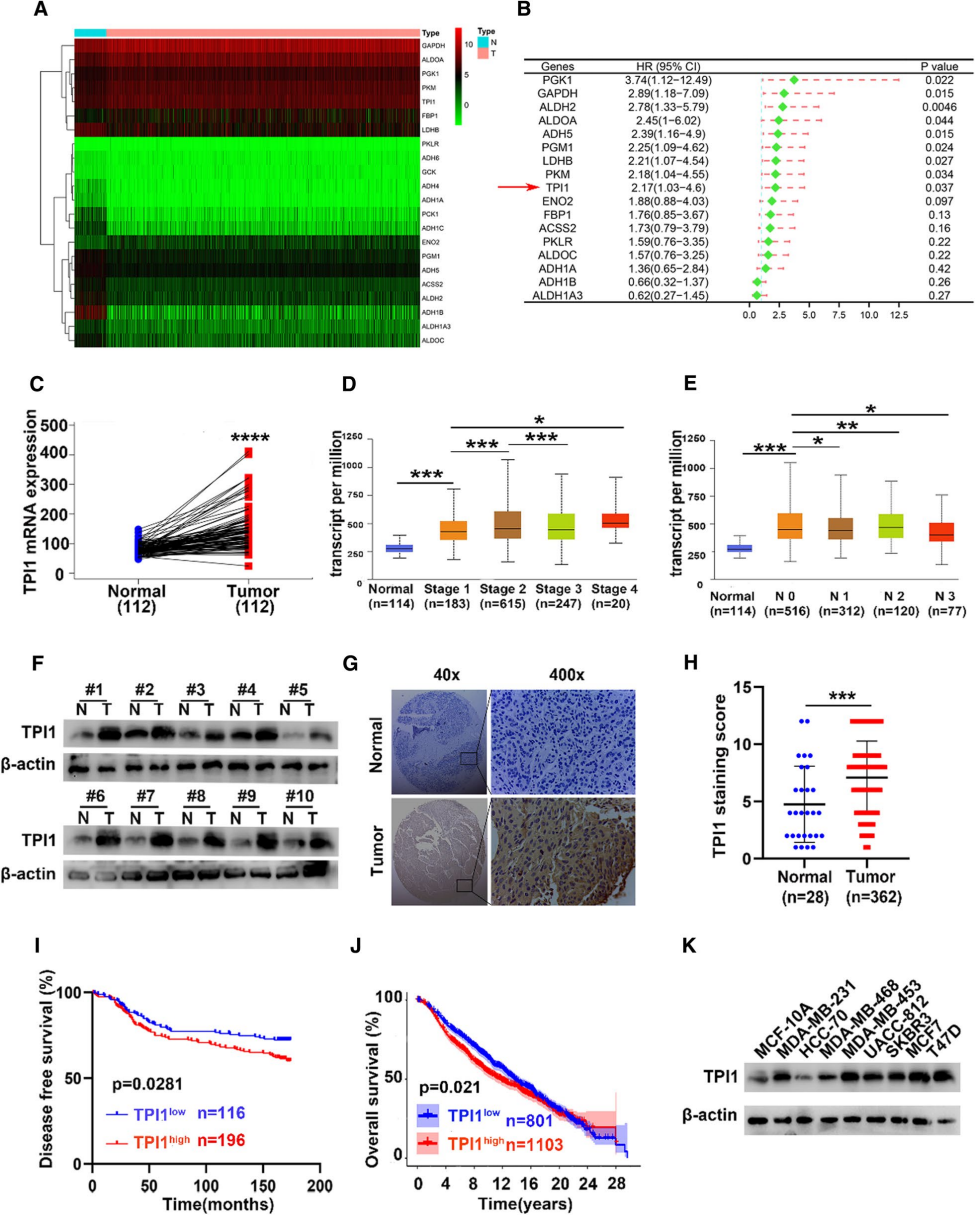
High expression of TPI1 positively correlates with poor prognosis in BRCA. **A** Expression of glycolysis and gluconeogenesis related genes in normal and tumor breast tissues. **B** Univariate Cox regression model was applied to calculate hazard ratios (HRs) of glycolysis-related genes from Tang (2018) dataset of Kaplan–Meier plotter database. **C** TPI1 gene expression in human BRCA tissues and matched normal tissues (n = 112) from TCGA. **D**, **E** Expression of TPI1 in BRCA based on individual clinical stages and lymph node stages (N) (TCGA). **F** TPI1 expression levels in fresh BRCA tumor (T) tissues and matched normal (N) tissues were examined by western blotting. **G** Representative image of IHC staining of TPI1 in BRCA tissue microarray. Magnification, ×40 and ×400. **H** TPI1 expression levels were compared in BRCA tumor tissue and normal tissue. **I**, **J** Kaplan–Meier curves to display relationships between TPI1 expression and DFS or OS in BRCA patients in Harbin Medical University Cancer Center (HMUCC) (**I**) and METABRIC database (**J**), respectively. **K** Western blotting analysis of TPI1 expression in MCF-10A cells and eight human BRCA cell lines. β-Actin served as a control. (*p < 0.05, **p < 0.01, ***p < 0.001, ****p < 0.0001)

To screen for potential oncogenes in glycolysis-related genes in BRCA, 62 glycolysis-associated genes were selected. Expression profile of these genes were compared between normal mammary and BRCA tumor tissues in TCGA. A heatmap display differential expression of 62 genes (Fig. 1A). Univariate Cox proportional hazards regression analysis was conducted to evaluate hazard ratios for overall survival (Fig. 1B) with KM plotter. Consequently, TPI1 was selected as a target gene, with high expression and risk for poor prognosis in BRCA.

TPI1 expression was examined in TCGA. Notably, TPI1 in primary BRCA was significantly higher than that in normal breast tissue (Additional file 1: Fig. S1A), as well as in 112 pairs of cancer and noncancerous adjacent tissues (Fig. 1C). TPI1 was positively associated with clinical stage (Fig. 1D) and lymphatic metastasis (Fig. 1E) of BRCA (in TCGA). In terms of human tissue samples, based on WB, TPI1 expression was significantly upregulated in 10 pairs of BRCA samples compared with the matched adjacent normal tissues (Fig. 1F). According to IHC from tissue array, TPI1 was expressed in cytoplasmic and nucleus of tumor cells, and significantly higher in BRCA samples than in normal tissues (Fig. 1G, H). The KM plotter demonstrated that patients with high TPI1 expression levels exhibited shorter disease-free survival (DFS) in the HMUCC cohort (*p* < 0.05, Fig. 1I) as well as curtailed OS in the METABRIC database (*p* < 0.05, Fig. 1J). In different cell lines, TPI1 expression was higher in BRCA cells than in normal mammary epithelial MCF-10A cells (Fig. 1K). These results revealed that TPI1 was a biomarker for poor prognosis in BRCA.

Next, association between TPI1 expression and clinicopathological parameters of 362 patients with BRCA was analyzed (Table 1). TPI1 expression in BRCA positively correlated with clinical stages (*p* = 0.002) and p53 level (*p* = 0.048). No significant association was observed between TPI1 expression and age, tumor size, lymph node status, histological grade, ER, PR, HER2, or KI67 (Table 1). Importantly, univariate and multivariate analyses indicated that TPI1 expression level, tumor size and lymph node status were independent indicators for BRCA prognoses (Table 2). Thus, TPI1 had potential clinical value as a predictive biomarker for diagnosis and prognosis in BRCA.

**Table 1:** Relationship of TPI1 expression with clinicopathologic features in 362 BRCA patients

Variables	Number of Patients		*P* (χ^2^)
	TPI1^low expression^	TPI1^high expression^	
Age (years)			0.516
≤ 40	27	37	
> 40	139	159	
Tumor size (cm)			0.059
≤ 2	58	92	
2–5	100	94	
> 5	8	10	
Lymph node status			0.504
Negative	88	97	
Positive	78	99	
Clinical stages			0.002**
I	29	58	
II	96	78	
III	41	60	
Grade			0.185
G1	8	14	
G2	118	148	
G3	11	6	
ER			0.596
Negative	57	58	
Positive	92	106	
PR			0.838
Negative	33	35	
Positive	115	129	
HER2			0.517
Negative	118	125	
Positive	30	38	
KI67			0.323
Negative	23	19	
Positive	122	140	
P53			0.048*
Negative	93	120	
Positive	51	40	

TPI1, scored > 7, was defined as high expression; ≤ 7, represented low expression. χ^2^ test was used for comparing groups with low and high TPI1 expression. n = 362

BRCA breast cancer

*p < 0.05 was considered statistically significant

**Table 2:** Univariate and multivariate Cox regression models of TPI1 expression in BRCA patients

Variables	Univariate Cox regression			Multivariate Cox regression		
	HR	95% CI	*P*	HR	95% CI	*P*
DFS						
Age (years)						
≤ 40 vs. > 40	1.157	0.717–1.868	0.551	–	–	–
TPI1 expression						
High vs. low	1.500	1.041–2.162	0.029*	1.461	1.009–2.114	0.045*
Tumor size (cm)						
≤ 2 vs. 2–5 vs > 5	2.116	1.543–2.901	0.000***	1.826	1.333–2.501	0.000***
Lymph node status						
N0 + N1 vs. N2 + N3	3.774	2.644–5.388	0.000***	3.186	2.207–4.601	0.000***
Grade						
G1 vs. G2 and G3	2.506	0.924–6.802	0.071	–	–	–

*DFS* disease-free survival, *HR* hazard ratio, *CI* confidence interval

### Knockdown TPI1 suppresses malignant phenotypes of BRCA in vitro

To explore biological functions of TPI1 in BRCA, co-expressed or related genes with TPI1 were obtained from Linkedomics, cBioportal, UALCAN, and GEPIA datasets. These genes were cross-referenced to obtain a cohort of 61 commonly co-expressed genes (Additional file 1: Fig. S1B). 61 genes were enriched and analyzed with Kyoto Encyclopedia of Genes and Genomes (KEGG). TPI1 was associated with cell cycle, glycolysis, ubiquitin-mediated proteolysis, and small cell lung cancer (Additional file 1: Fig. S1C). Furthermore, gene ontology analysis indicated that TPI1 correlated with cell division, cell cycle G1/S phase transition, nucleocytoplasmic transport, ubiquitin-like protein ligase binding, and cadherin binding (Additional file 1: Fig. S1D–F). These results suggested that TPI1 played an important role in modulating proliferation and glycolysis in cancer.

**Fig. 2:**
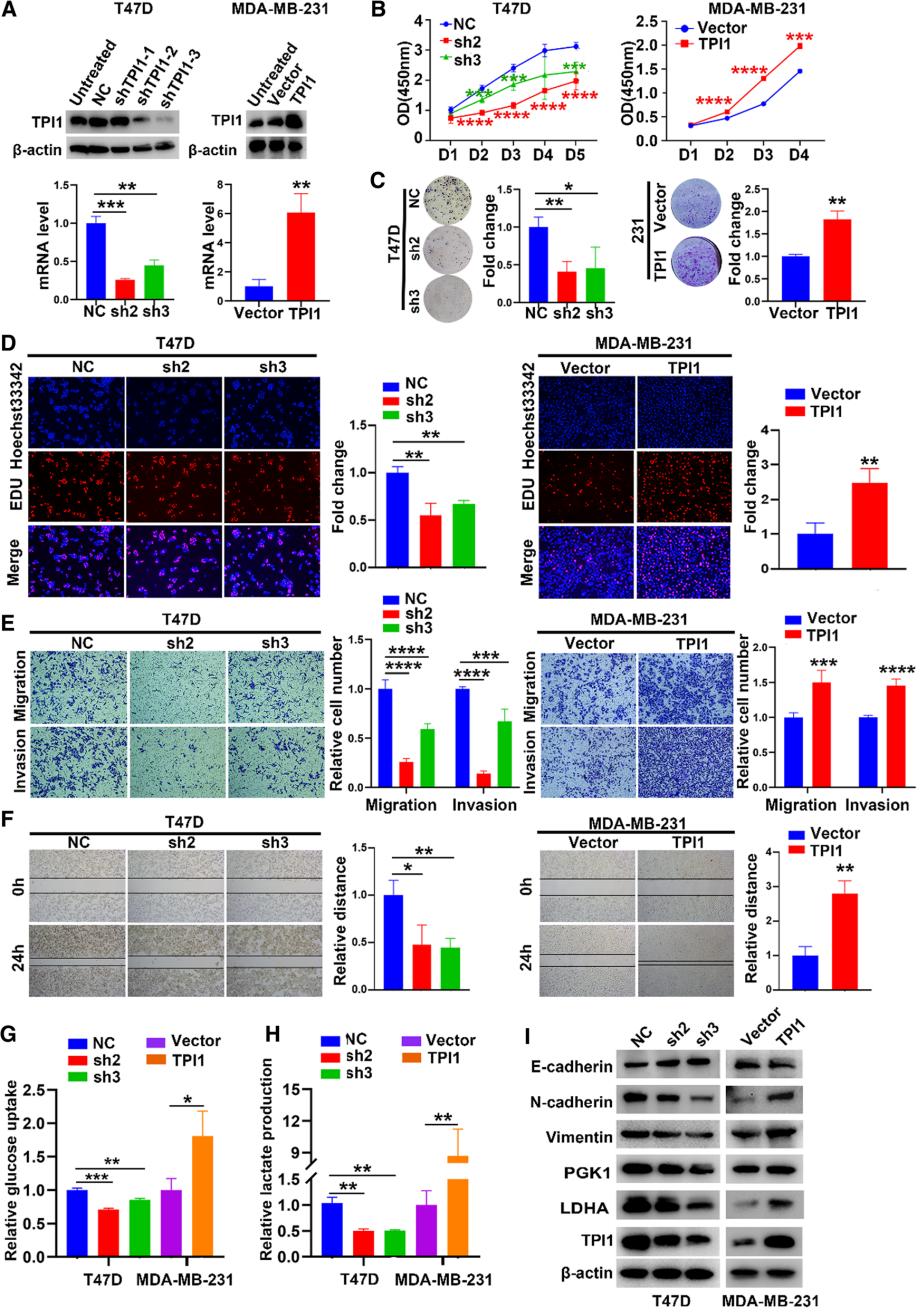
TPI1 promotes proliferation, migration, invasion and aerobic glycolysis in BRCA in vitro. **A** TPI1 protein and mRNA expression levels were determined in T47D cells (left panel) by stably expressing control vector or shTPI1 (shTPI1-1, 2, 3, respectively) and in MDA-MB-231 cells (right panel) by stably expressed empty vector or TPI1, shTPI1-2 and shTPI1-3 (sh2, sh3) were relatively effective at knocking down. β-actin served as a control. **B** Cellular viability of T47D/sh2, sh3 and T47D/NC (left panel), MDA-MB-231/TPI1 (right panel) and vector control were analyzed by CCK-8 assays. **C** Colony forming assays were conducted in T47D/sh2, sh3 and T47D/NC (left panel), MDA-MB-231/TPI1 (right panel) and vector control. **D** Edu assays were conducted in T47D (left panel) and MDA-MB-231 (right panel) cells. **E** Transwell assays were conducted to assess cell migration and invasion after knockdown or overexpression of TPI1, respectively, in T47D (left panel) and MDA-MB-231 (right panel) cells compared with corresponding vector cells. **F** Wound healing assays were performed in T47D (left panel) and MDA-MB-231 (right panel) cells. **G**, **H** Relative glucose uptake (**G**) and lactate production (**H**) were determined in T47D and MDA-MB-231 cells. **I** Western blot analysis of indicated proteins in TPI1 knockdown and TPI1 overexpression cells, respectively. β-Actin served as a control. Experiments were performed at least three times. Data were presented as the means ± SEMs (*p < 0.05, **p < 0.01, ***p < 0.001, ****p < 0.0001)

TPI1 shRNA was stably transfected into T47D cells with high endogenous TPI1 expression. A vector encoding for human TPI1 gene was stably transfected into MDA-MB-231 cells with low endogenous TPI1 expression (Fig. 1K). Transfection efficiency was verified by WB and qPCR (Fig. 2A). ShTPI1-2 (sh2) and shTPI1-3 (sh3) had a higher efficiency for knockdown. Interestingly, cell proliferation and colony formation were declined more significantly in T47D/sh2 and T47D/sh3 cells than in cells expressing vector control as demonstrated by CCK-8, colony-forming and Edu tests (Fig. 2B–D). Moreover, silencing of TPI1 reduced invasion and migration in T47D cells (Fig. 2E, F) as well as decreased glucose uptake and lactate production (Fig. 2G, H). Consistently, N-cadherin, vimentin, phosphoglycerate kinase 1 (PGK1) and lactate dehydrogenase A (LDHA) were downregulated whereas E-cadherin was upregulated (Fig. 2I), which could be reversed by upregulation of TPI1 in MDA-MB-231 cells (Fig. 2B–I). These results implied that downregulation of TPI1 might inhibit BRCA aggressiveness in vitro.

### PI3K/AKT/mTOR pathway is necessary to promote EMT-related metastasis and aerobic glycolysis of BRCA

**Fig. 3:**
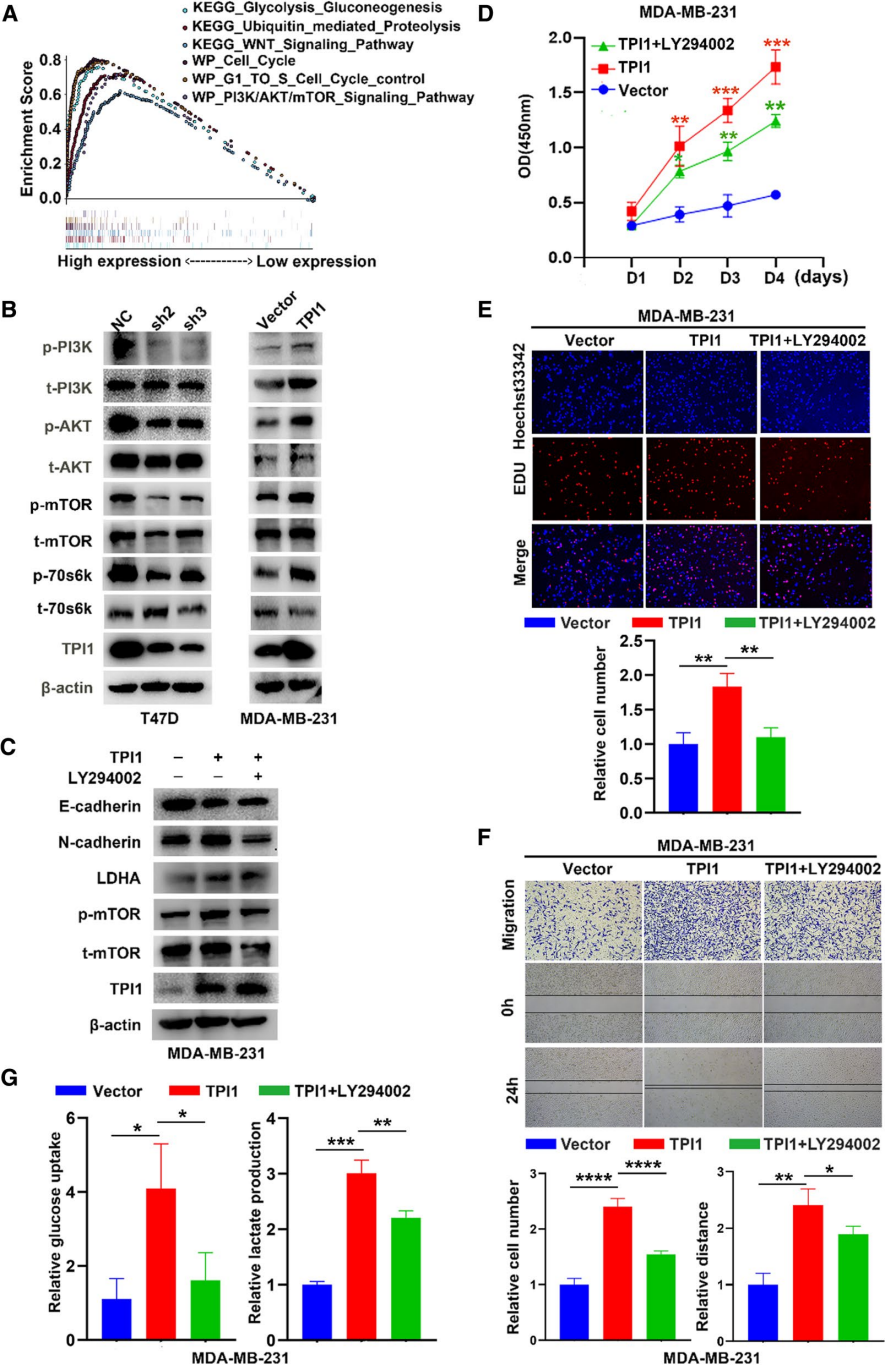
PI3K/AKT/mTOR signaling is essential for TPI1 mediated EMT and aerobic glycolysis. **A** TPI1 enriched in GSEA. NES, normalized enrichment score. Positive relationship of TPI1 levels with PI3K/AKT/mTOR, WNT signal, glycolysis gluconeogenesis and ubiquitin mediated proteolysis. **B** Western blot was used to detect levels of key signal transduction proteins in T47D and MDA-MB-231 cells. β-Actin served as a control. **C** Western blot was applied to explore expression of E-cadherin, N-cadherin, LDHA, p-mTOR and t-mTOR with LY294002 (PI3K inhibitor, 50 μM). β-Actin served as a control. **D** The CCK-8 assay was assessed in MDA-MB-231/TPI1 and MDA-MB-231/TPI1 after incubation with LY-294002. **E** The Edu assay was performed in those indicated cells. **F** Migration and wound-healing assays were performed to investigate mobility of TPI1 in MDA-MB-231 cells treated with or without LY-294002. **G** Relative glucose uptake (left panel) and lactate production (right panel) were determined in TPI1-overexpressing cells treated with or without LY-294002 (*p < 0.05, **p < 0.01, ***p < 0.001)

To understand how TPI1 influences BRCA progression, a gene set enrichment analysis (GSEA) was performed by dividing samples into two groups based on TPI1 expression in the TCGA database. TPI1 was positively related to common pathways in BRCA, such as PI3K/AKT/mTOR, WNT signal and pluripotency, glycolysis gluconeogenesis and ubiquitin-mediated proteolysis (Fig. 3A). Notably, upregulation of TPI1 activated key proteins of PI3K/AKT/mTOR in MDA-MB-231 cells, whereas its downregulation had an opposite effect (Fig. 3B). Next, LY294002 (PI3K/AKT pathway inhibitor) was used to explore if TPI1 mediated BRCA progression through PI3K/AKT/mTOR pathway. The expression of N-cadherin, LDHA and p-mTOR were reduced when incubated with LY294002 (Fig. 3C). Exposure to LY294002 led to a significant decrease in CCK-8 (Fig. 3D) and Edu (Fig. 3E) assays among TPI1-overexpression cells compared with untreated cells. Furthermore, in transwell and wound-healing assays, EMT-related migration was partly reversed in MDA-MB-231/TPI1 cells treated with LY-294002 (Fig. 3F). Similarly, glucose consumption and lactate production were partially reversed by LY-294002 (Fig. 3G). Taken together, upregulation of TPI1 activated PI3K/AKT/mTOR pathway, which in turn promoted EMT and aerobic glycolysis in BRCA cell.

### TPI1 activates AKT/mTOR pathway by stabilizing CDCA5 expression

**Fig. 4:**
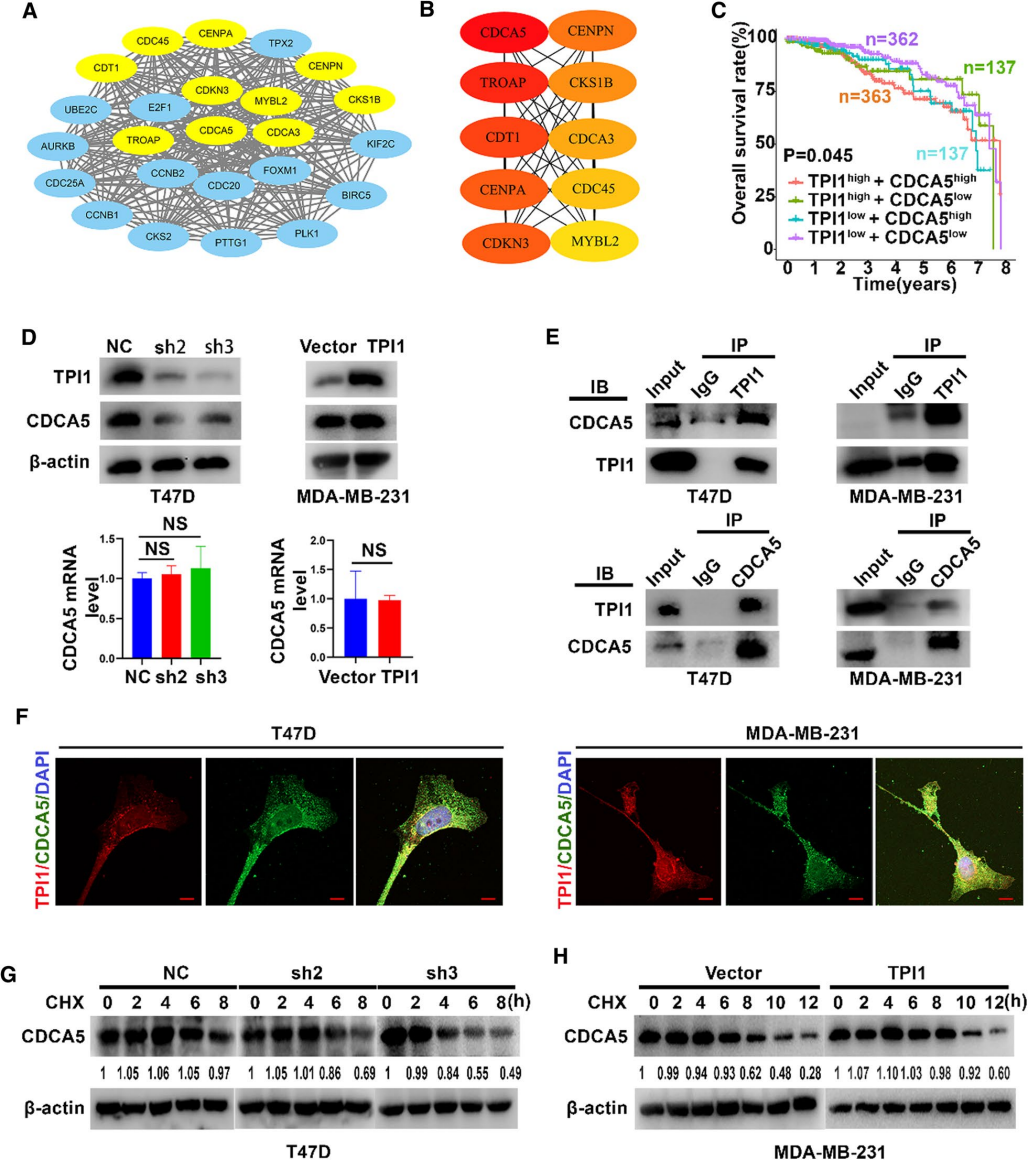
TPI1 interacts with CDCA5 and stabilizes CDCA5 protein. **A** Clustering analysis of 61 genes co-expressed with TPI1 by STRING tools. Relatively important module was highlighted with Cytoscape tool. **B** Hub genes were identified using cyto-Hubba tool kits in Cytoscape. **C** Kaplan–Meier plotter described relationship of differentially expressed TPI1/CDCA5 with OS (based on log-rank test). High levels of TPI1 and CDCA5 were associated with shortened OS in TCGA. **D** Western blot and qRT-PCR used to quantify expression of CDCA5 in TPI1 knockdown vs. overexpression cells. CDCA5 protein level, but not mRNA level was changed (NS represented no statistical significance). **E** Interaction between endogenous TPI1 and CDCA5. IP lysates were used to analyze expression of TPI1 and CDCA5 by western blot. **F** Immunofluorescence used to verify formation of TPI1/CDCA5 complex in those indicated cells, Scale bar, 10 μm. **G** T47D cells stably expressing control, sh2 and sh3 were treated with CHX (200 μg/ml) for indicated time points (0, 2, 4, 6, 8 h, respectively). Cells were lysed and examined by western blot. **H** MDA-MB-231 cells expressing vector and TPI1 were treated with CHX (200 μg/ml) for indicated time points (0, 2, 4, 6, 8, 10, 12 h, respectively). Cells were lysed and examined by western blot. β-Actin served as a control. The numbers represented relative expression of CDCA5 in **G** and **H**. NS represented no statistical significance

To illustrate underlying mechanisms of how TPI1 activates PI3K/AKT/mTOR signaling pathway, co-expressed 61 genes were constructed into a protein–protein interaction network using the STRING database. The top 10 candidates were identified as potential hub genes according to individual degree score generated by Cyto-Hubba plug-in (top 10 nodes ranked by DMNC). CDCA5 was ranking #1, consistent with its enrichment in the top modules as analyzed by MCODE (Fig. 4A, B). In addition, patients with high expression of TPI1 and CDCA5 had poorer OS (Fig. 4C) from TCGA database. CDCA5 might be involved in regulating TPI1.

CDCA5 expression was increased in breast tumor tissues than normal breast tissues in TCGA (Additional file 1: Fig. S2A). Furthermore, patients with high levels of CDCA5 expression had reduced OS (*p* = 0.031) in TCGA (Additional file 1: Fig. S2B). To determine potential functions of CDCA5 in BRCA, CDCA5 plasmid was transfected into T47D cells. Overexpression of CDCA5 promoted proliferation, migration, invasion, glucose uptake, and lactate production compared to vector control (Additional file 1: Fig. S2C–G). In addition, overexpression of CDCA5 upregulated p-PI3K, p-AKT, p-mTOR, related markers of EMT, and glycolysis as shown by WB (Additional file 1: Fig. S2H). These data indicate that upregulation of CDCA5 might activate PI3K/AKT/mTOR pathway and promote BRCA cell malignant behaviors.

**Fig. 5:**
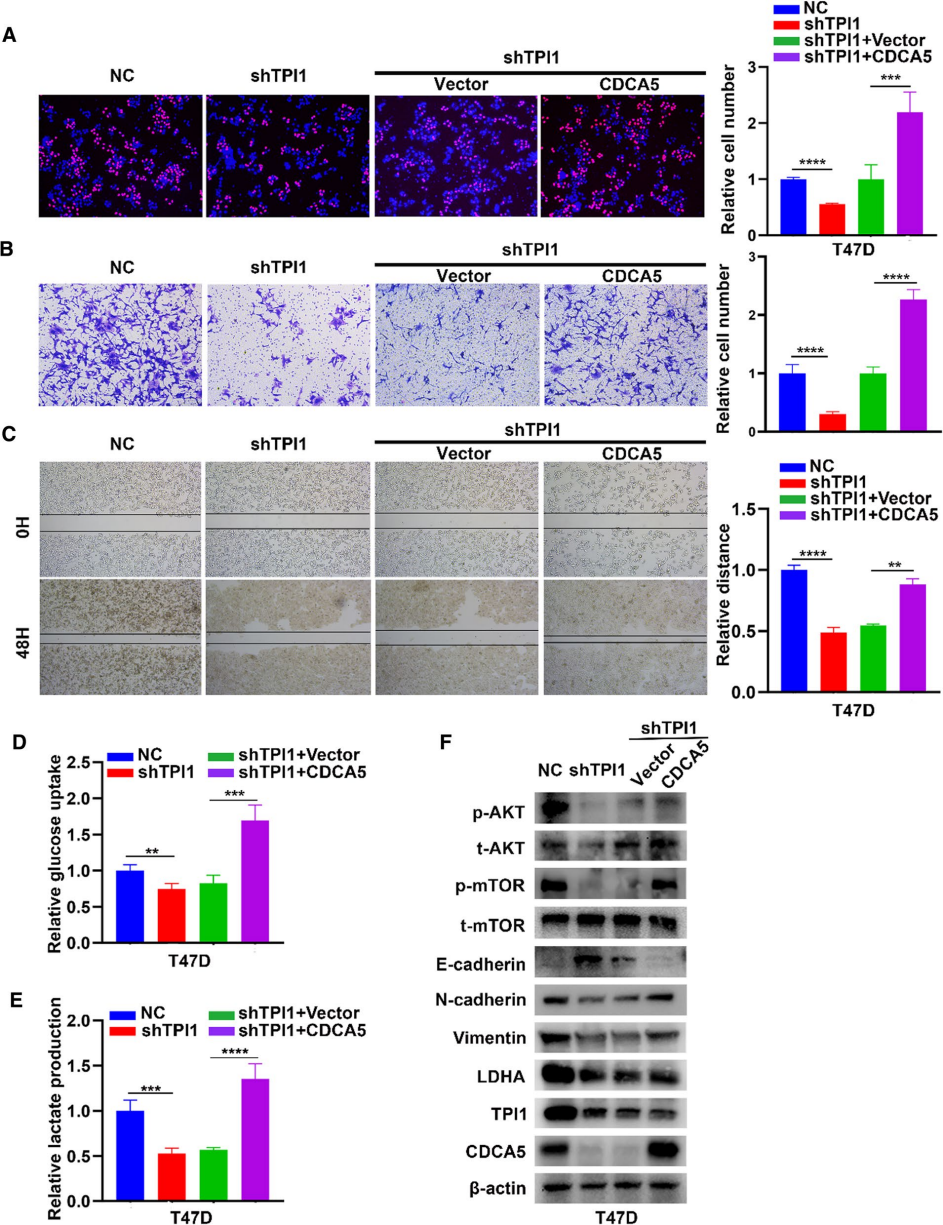
CDCA5 is essential for TPI1-mediated proliferation, EMT and aerobic glycolysis. **A** Overexpressing CDCA5 reversed reduction of proliferation owing to stably silencing TPI1 in T47D cells. **B**, **C** Rescue assays for migration (**B**) and wound healing (**C**) were performed after overexpressing CDCA5 in T47D/shTPI1 cells. **D**, **E** Rescue assays for glucose uptake (**D**) and lactate production (**E**) in indicated cells. **F** Western blot analysis of AKT, p-AKT, mTOR, p-mTOR, EMT markers, and LDHA expression in T47D/shTPI1 treated with or without overexpression of CDCA5. β-Actin served as a control (**p < 0.01, ***p < 0.001, ****p < 0.0001)

Moreover, TPI1 knockdown led to downregulation of CDCA5 protein but not mRNA. TPI1 overexpression obtained consistent results (Fig. 4D). Thus, CDCA5 potentially interacted with TPI1 as a downstream target of TPI1. Subsequent Co-IP and IF assays confirmed an interaction between TPI1 and CDCA5 proteins (Fig. 4E, F). Finally, a CHX assay indicated that the half-life of CDCA5 protein was shortened after TPI1 knockdown in T47D cells (Fig. 4G). In contrast, TPI1 overexpression in MDA-MB-231 cells prolonged the half-life of CDCA5 (Fig. 4H). Therefore, TPI1 enhanced the stability of CDCA5 protein via interaction. To determine whether CDCA5 participated in TPI1-mediated BRCA progression, CDCA5 was transfected into T47D/shTPI1 cells. Based on Edu, overexpression of CDCA5 reversed inhibitory effect of TPI1 knockdown on proliferation in T47D cells (Fig. 5A). Overexpression of CDCA5 based on TPI1 knockdown increased migration to more extent than in shTPI1 cells (Fig. 5B, C). Glucose uptake (Fig. 5D) and lactate production (Fig. 5E) were reversed after upregulation of CDCA5 in T47D/shTPI1 cells. These results were validated with pivotal protein markers in WB (Fig. 5F). Together, TPI1 stabilized CDCA5 to activate PI3K/AKT/mTOR pathway.

### TPI1 facilitates BRCA progression in vivo

To evaluate potential functions of TPI1 in tumor progression of BRCA in vivo, MDA-MB-231 cells were stably transfected with vector-luciferase (vector) and overexpressing TPI1-luciferase (TPI1) using lentivirus system. T47D cells were stably transfected with control (NC) or TPI1shRNA (shTPI1) with lentivirus system. Cells were subcutaneously injected into the left axillary regions of nude mice. The growth of tumors was monitored over 7–35 days after implantation before removing for analysis.

**Fig. 6:**
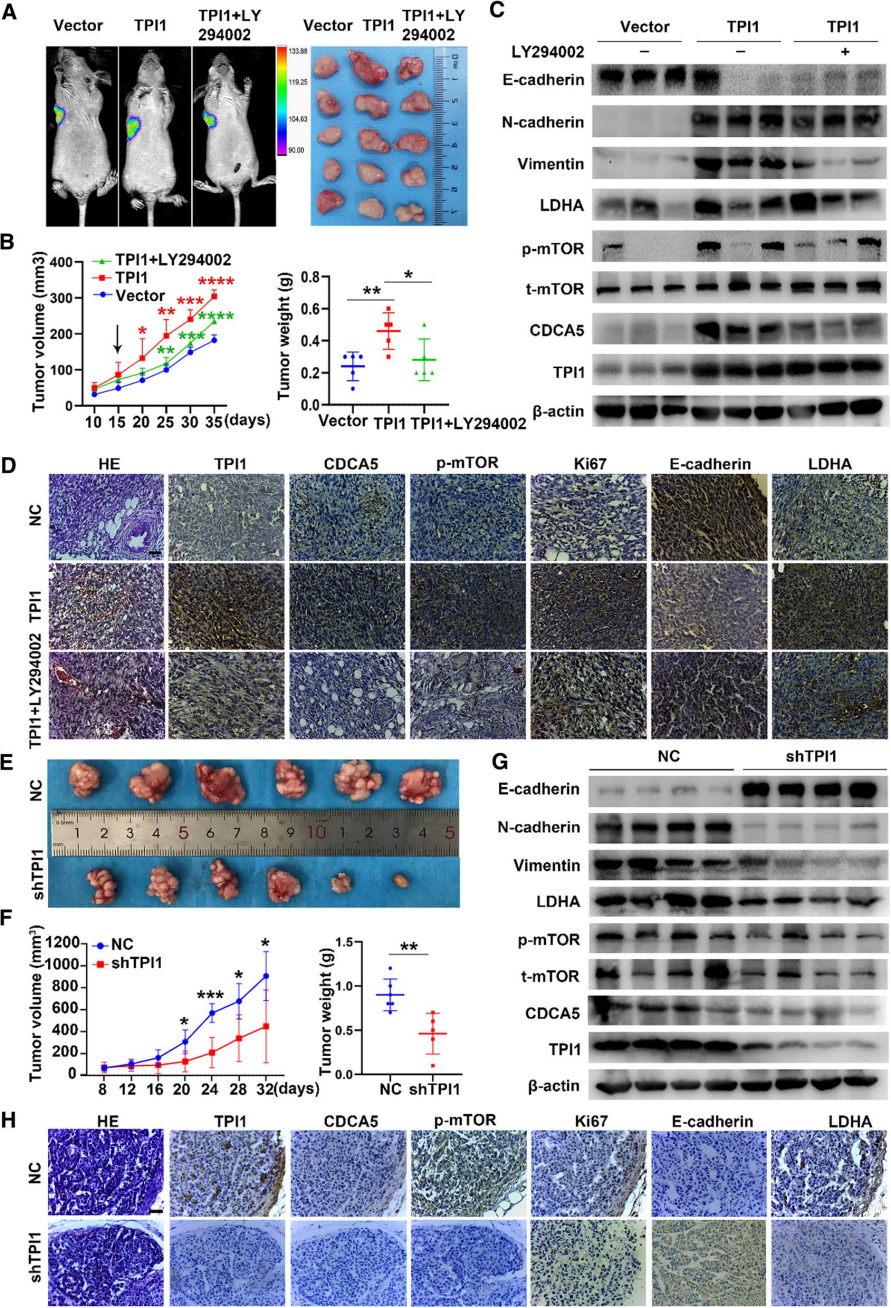
TPI1 facilitates breast cancer progression in vivo. **A**–**D** MDA-MB-231 luciferase cells stably expressing vector or TPI1 were transplanted into the left axillary regions of nude mice. A group of nude mice were randomly selected to receive MDA-MB-231 cells and treated with LY294002 (75 mg/kg), once every 3 days after primary tumor formation (n = 5/group). Representative bioluminescence photograph and resulting tumors on day 35 (**A**). Tumor size was measured at 5-day intervals after primary tumor formation (**B**, left panel). Tumor weight was measured in different groups (**B**, right panel). Western blot analysis of related markers in mouse tumor tissues (**C**). β-Actin served as a control. Representative IHC images of mouse tumor tissues with TPI1, CDCA5, p-mTOR, Ki67, E-cadherin and LDHA (**D**). Scale bar, 20 μm. **E**–**H** T47D/vector cells and stable TPI1-knockdown (shTPI1) cells were subcutaneously injected. The photograph of tumors on day 32 (**E**), tumor growth curves on day 8–32 (**F**, left panel) and tumor weight (**F**, right panel) were presented (n = 6/group). Western blot analysis of indicated markers from harvested mouse tumor tissues (**G**). β-Actin served as a control. Representative IHC images of mouse tumor tissues (**H**). Scale bar, 20 μm (*p < 0.05, **p < 0.01, ***p < 0.001, ****p < 0.0001)

Based on representative bioluminescence images, luciferase activity of overexpression of TPI1 was significantly higher than in NC group, whereas fluorescence was decreased after administration of LY294002 (Fig. 6A, left panel). Tumor size exhibited the same trend (Fig. 6A, right panel). The tumors growth curve (Fig. 6B, left panel) and tumor weight (Fig. 6B, right panel) were higher for TPI1 group than vector control. However, LY-294002 reversed TPI1-mediated promotion of tumor formation (Fig. 6B). Finally, overexpression of TPI1 increased expression of N-cadherin, vimentin, LDHA, p-mTOR, and CDCA5, whereas reduced E-cadherin in tumor tissues as demonstrated by WB. This trend was reversed by LY-294002 (Fig. 6C). In addition, based on IHC, expression levels of CDCA5, Ki67, p-mTOR, and LDHA were higher in tumor tissue generated by TPI1 overexpression cells than vector control; E-cadherin was lower in these tumors. LY294002 could reverse this trend (Fig. 6D). When TPI1 was knocked down, tumor size (Fig. 6E) was reduced in shTPI1 group than NC group. Tumor growth curves (Fig. 6F, left panel) and tumor weight (Fig. 6F, right panel) were inhibited in shTPI1 group compared to NC group. Finally, related markers were validated with WB (Fig. 6G) and IHC (Fig. 6H). Thus, TPI1 activated PI3K/AKT/mTOR pathway, which in turn promoted BRCA progression in vivo.

### Sequestosome-1 (SQSTM1)/p62 interacts with TPI1 and promotes its ubiquitination in BRCA cells

**Fig. 7:**
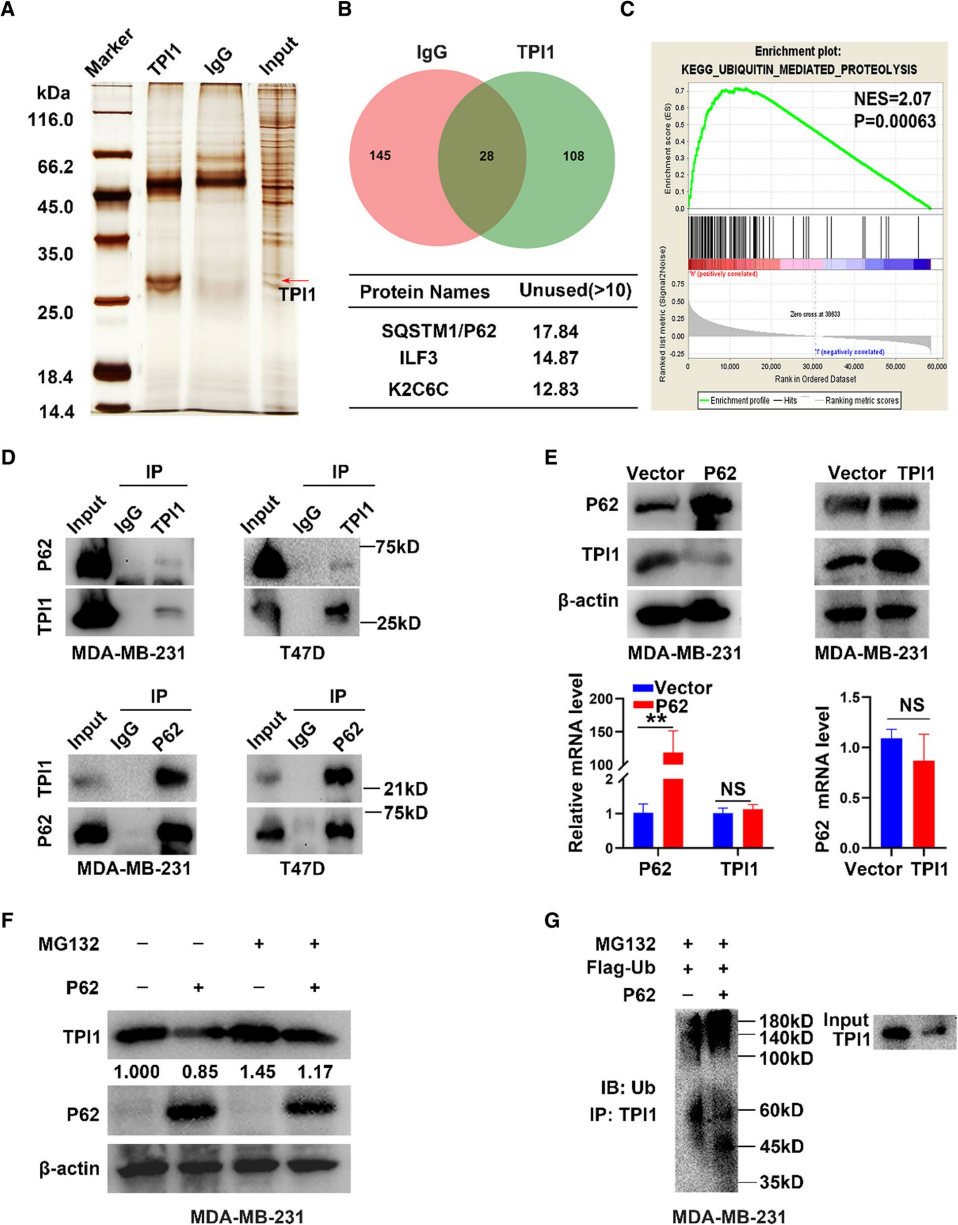
SQSTM1/P62 interacts with TPI1 to down-regulate TPI1 protein level in BRCA. **A** Silver staining assay identified TPI1-specific bands. **B** Mass spectrometry identified 3 mainly interacting proteins. **C** GSEA analysis showed TPI1 involved in ubiquitination degradation. **D** IP analysis of MDA-MB-231 and T47D cells with antibodies against TPI1 and P62. P62 and TPI1 were analyzed by immunoblotting. **E** Western blot and qRT-PCR used to quantify expression of TPI1 and P62 in MDA-MB-231 cells. Overexpression of P62 down-regulated TPI1 at protein level, but not at mRNA level. NS represents no statistical significance. **F** MDA-MB-231 cells were transfected with P62 plasmid and treated with or without MG132 (10 μM) for 6 h. Cell lysates were analyzed by western blot with indicated antibodies. **G** Ubiquitination assay to examine effects of P62 on TPI1 ubiquitination. Flag-Ub and p62 plasmid were co-transfected into MDA-MB-231 cells, then incubated with MG132 for 6 h

To elucidate underlying mechanisms by which TPI1 stabilizes CDCA5, protein partners of TPI1 were identified in MDA-MB-231 cells through silver staining assay with IP lysates (Fig. 7A). TPI1 and IgG protein bands were subjected to mass spectrometry (MS), which identified 28 potential TPI1-interacting proteins based on unique peptides (> 1) with repeated appearance (Fig. 7B, upper panel).

Interestingly, SQSTM1/P62 was the most reliable binding protein according to MS (Fig. 7B, lower panel). The C-terminal ubiquitin-associated domain of SQSTM1/P62, as a ubiquitin-associated protein, binds to ubiquitin-conjugated proteins. Simultaneously, based on KEGG (Additional file 1: Fig. S1C) and GSEA (Fig. 7C), TPI1 might be involved in ubiquitin-mediated proteolysis. Thus, P62 might promote TPI1 ubiquitination. Then, interaction between TPI1 and P62 was verified by co-IP assay (Fig. 7D). TPI1 had no effect on P62 mRNA and protein levels (Fig. 7E, right panel). However, TPI1 protein level was significantly reduced when P62 was overexpressed, even though TPI1 mRNA level was unchanged (Fig. 7E, left panel). Notably, decreased TPI1 protein level in MDA-MB-231 cells overexpressing P62 could be recovered partially by being treated with MG132 (Fig. 7F). Finally, overexpression of P62 increased TPI1 ubiquitination (Fig. 7G). Together, P62 might be involved in degradation of TPI1 through ubiquitination.

### TPI1 correlates with CDCA5 and p-mTOR in clinical BRCA specimens

**Fig. 8:**
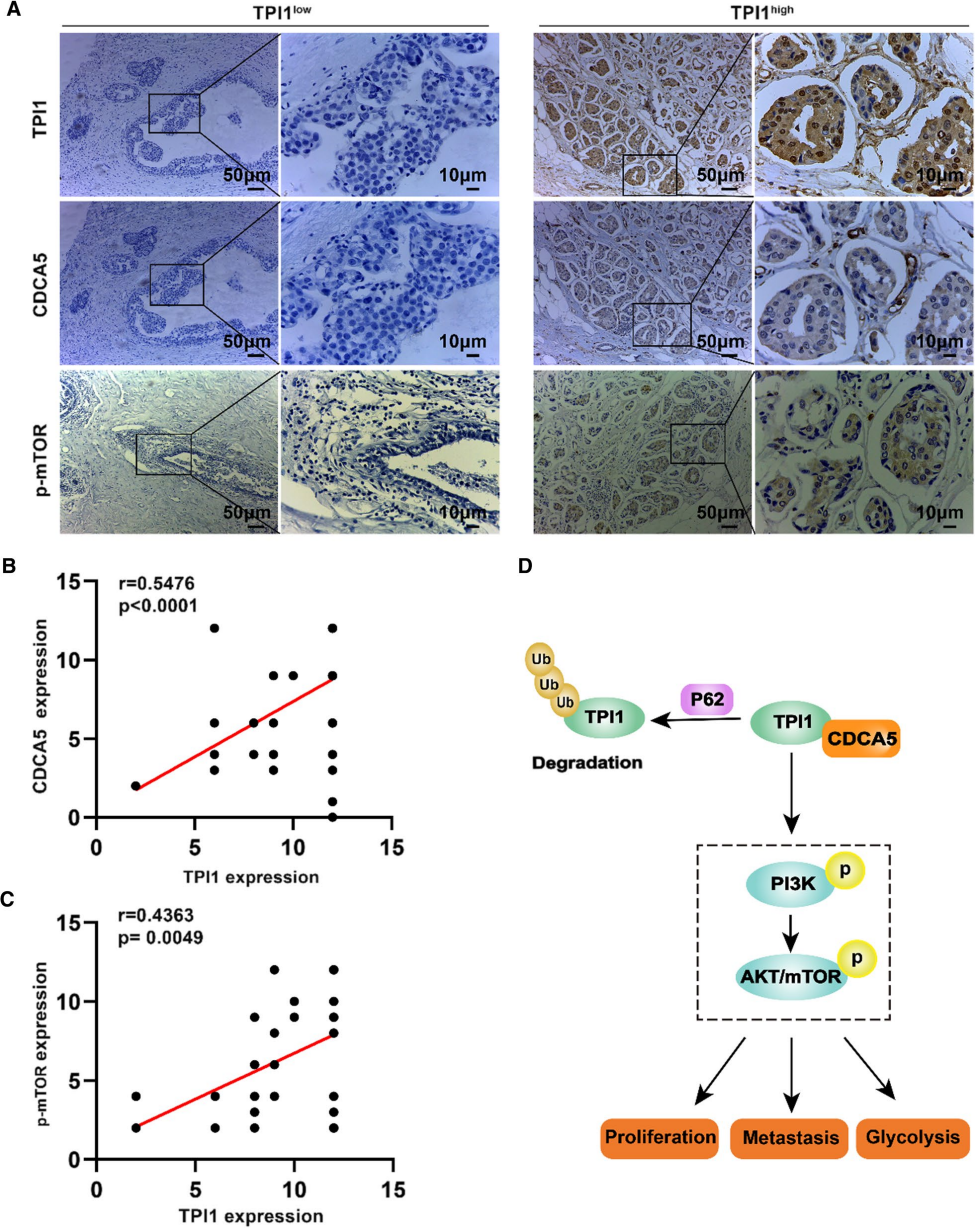
TPI1 correlates with CDCA5 and p-mTOR in BRCA tissues from patients. **A** Representative images of immunohistochemical staining for TPI1, CDCA5 and p-mTOR in BRCA samples from patients with TPI1 high (score > 9) and low expression (score ≤ 9), respectively. Scale bar, 50 μm/10 μm. **B**, **C** Correlation of TPI1 with CDCA5 (**B**) and p-mTOR (**C**) in clinical BRCA tissues (n = 50). **D** Hypothesized roles of TPI1 in BRCA. The r and p values were from Pearson’s correlation

To examine the relationship of TPI1 with CDCA5, and p-mTOR in human BRCA, IHC staining was performed on continuous tissue sections from 50 randomly selected BRCA patients. Consistent with findings in cell lines and mice models, the expression of TPI1 positively correlated with CDCA5 (r = 0.5476, *P* < 0.0001, Fig. 8A, B) and p-mTOR (r = 0.4363, *P* = 0.0049, Fig. 8A, C) in BRCA tissue specimens. Therefore, TPI1 might be a novel prognostic biomarker and a therapeutic target for BRCA.

## Discussion

In this study, TPI1 has been identified as a marker of poor prognosis in BRCA. By stabilizing CDCA5 protein, TPI1 activates PI3K/AKT/mTOR pathway, thereby enhancing tumor progression and glycolysis. In addition, P62 interacts with TPI1 and regulates protein stability. Our study, for the first time, demonstrates the roles of TPI1 in promoting breast cancer progression, and provides a new strategy for targeted glycolysis therapy.

High morbidity and mortality rates of breast cancer urge discovery for new effective therapy [2, 26]. Dysregulated metabolism is a hallmark of cancer [27]. Aerobic glycolysis is notable, in which cancer cells preferentially depend on aerobic glycolysis to obtain energy, even in the presence of abundant oxygen [6]. Aerobic glycolysis promotes unrestricted growth and metastasis of cancer cells [11]. Therefore, to target glycolysis is a promising therapeutic strategy for breast cancer. In the present study, TPI1 predicts poor prognosis in BRCA. Mechanistically, through stabilizing CDCA5, TPI1 activates PI3K/AKT/mTOR pathway, which in turn promotes breast cancer metastasis and glycolysis. Moreover, TPI1 undergoes ubiquitin-dependent proteasome degradation in the presence of P62 in BRCA cells (Fig. 8D).

In order to better understand regulatory mechanisms of aerobic glycolysis in BRCA, key genes were identified with KEGG. Multiple bioinformatics analyses identified TPI1 as a crucial glycolysis enzyme, which is overexpressed in BRCA and predicts poor prognosis. However, TPI1 was previously proposed as a tumor suppressor gene. In BRCA, TPI1 has been upregulated at cellular and tissue level. We noted that TPI1 was low expressed in triple negative breast cancer cell lines (HCC70 and MDA-MB-468). Somatic mutation frequency and gene expression level have a strong correlation in cancers [28]. Just as BRCA1 mutation is more common in triple negative breast cancer, whereas BRCA2 mutations are more common in Luminal B breast cancer [29]. Mutations at the dimer interface of TPI1 have been shown to lead to protein degradation and reduced TPI1 protein expression [30]. TPI1^E104D^ is one of the common mutation sites of TPI1 [31]. In addition, protein misfolding and accumulation of toxic substances may also contribute to reduced TPI1 expression [32]. The expression of TPI1 was not associated with ER/PR and HER2 molecular typing related genes in our breast cancer tissue samples. So, we cannot conclude that TPI1 has different effects on different molecular subtypes of breast cancer. In our study, high expression of TPI1 is positively associated with clinical stages in 362 BRCA samples. Importantly, univariate and multivariate models indicate that TPI1 is an independent indicator for prognosis of BRCA patients. Thus, TPI1 may be a potential biomarker in BRCA.

TPI1 expression is higher in tissue and plasma of lung cancer patients than in cancer-free humans [17]. Based on in vitro model and serological proteome, TPI1 may be assumed as a potential biomarker in lung carcinoma. On the contrary, TPI1 inhibits proliferation and metastasis via β-catenin and p53 signaling in HCC [23]. However, no studies have explored if TPI1 links to oncogenic phenotypes in BRCA. For the first time, we demonstrate that TPI1 promotes proliferation, migration, invasion, and glycolysis both in vitro and in vivo. Thus, TPI spurs malignant phenotypes in BRCA cells.

Metastasis causes as much as 90% of cancer-associated mortality in general [33–35], as a leading cause of BRCA deaths [36]. EMT is a major mechanism leading to tumor invasion and metastasis [37]. Meanwhile, PI3K/AKT/mTOR is a key pathway regulating proliferation, metastasis, and glycolysis [38, 39]. Activation of AKT can activate pro-EMT transcriptional factors, directly or indirectly, to stimulate EMT process and induce pro-metastatic molecules, resulting in tumor metastasis [40]. In addition, glycolytic enzyme PGK1 [37] and enolase 1 (ENO1) [11] promoted malignant phenotypes through AKT/mTOR pathway. Interestingly, upregulated TPI1 might activate PI3K/AKT/mTOR pathway based on GSEA. Meanwhile, expression of genes related to EMT and glucose metabolism were increased after overexpressing TPI1. Accordingly, knockdown TPI1 had the opposite effects. Treatment with LY294002 (PI3K pathway inhibitors) reversed phenotypes induced by TPI1 overexpression, demonstrating that TPI1 could regulate proliferation, EMT-related metastasis, and glucose metabolism through PI3K/AKT/mTOR pathway.

To elucidate how TPI1 regulates the PI3K/AKT/mTOR pathway, CDCA5 was identified as an important regulatory factor. CDCA5, also known as sororin, is a key player in sister chromatid cohesion and separation, and identified as a substrate of anaphase-promoting complex [41, 42]. Multiple studies have revealed that CDCA5 is overexpressed in various cancers, such as lung cancer, oral squamous cell carcinoma, gastric cancer, and HCC. CDCA5 was overexpressed in bladder cancer tissues and activated PI3K/AKT/mTOR pathway [41]. In HCC, CDCA5 promoted oncogenesis via AKT [43]. Similarly, CDCA5 is an indicator of poor prognosis based on BRCA TCGA data. Moreover, overexpression of CDCA5 significantly enhances proliferation, migration, and glycolysis by activating PI3K/AKT/mTOR pathway. Subsequently, co-IP and IF analyses confirmed interaction between TPI1 and CDCA5 protein, as upregulated TPI1 stabilized CDCA5 protein by CHX assay. After CDCA5 plasmid was transferred into T47D/shTPI1 cells, proliferation, migration and glycolysis were strengthened. Furthermore, TPI1 activates PI3K/AKT/mTOR pathway by stabilizing CDCA5 expression. Consistently, Ser209 phosphorylation on CDCA5 protein by ERK promoted growth or survival of lung cancer cells [44]. However, how TPI1 affects CDCA5 is still unclear, which should be elucidated in future studies.

Notably p62 was identified as a direct regulator of TPI1 by silver staining and mass spectrometry. The interaction between TPI1 and p62 was verified by co-IP assay. P62 contains several structural motifs, which may participate in forming multimeric signaling complexes. The C-terminal ubiquitin-associated domain of P62 binds to ubiquitin-conjugated proteins [45], while its N-terminal PB domain targets proteasome. Thus, P62 can act as a cargo receptor to promote proteasome degradation of ubiquitin proteins [46]. P62 promoted the ubiquitination of Tensin-2 through PB1 domain, leading to proteasome mediated degradation [47]. P62 also induced the ubiquitination and degradation of glycolytic enzyme 6-phosphofructo-2-kinase [34, 46]. Consistent with our results, overexpression of P62 decreased the protein expression of TPI1 through ubiquitination degradation. P62 may participate in the proteasome degradation of TPI1 as a cargo receptor. P62 is highly expressed in breast cancer. However, in our study, TPI1 is overexpressed in breast cancer, while p62 promotes degradation of TPI1. P62 [34] and TPI1 are expressed differently in different molecular types of breast cancer. The expression of P62 was relatively high in MDA-MB-231 cell [48], while the expression of TPI1 was relatively low. Thus, P62 induced ubiquitination of TPI1 in MDA-MB-231 cells. However, which fragment of P62 mediates its interaction with TPI1, and whether this phenomenon occurs in different molecular types remain to be explored. In short, we have only reported preliminary results on the relationship between P62 and TPI1 in this article, and we will continue in-depth studies in the future.

## Conclusions

In summary, TPI1, a glycolysis-associated gene, is overexpressed in BRCA. TPI1 promotes proliferation, metastasis and glycolysis by activating CDCA5-mediated PI3K/AKT/mTOR pathway both in vitro and in vivo. Moreover, TPI1 undergoes ubiquitin-dependent proteasome degradation in the presence of P62 in BRCA cells. To our knowledge, this is the first report on molecular mechanisms of TPI1 in BRCA. Our study demonstrates that TPI1 may be a potential prognostic biomarker and therapeutic target for BRCA. TPI1-targeting inhibitors and their combined pathway inhibitors for BRCA are also our future research direction.

## Supplementary Information

**Additional file 1: Table S1.** Antibodies were used to IHC. **Table S2.** Sequences of the interference TPI1 are the followings. **Table S3.** Antibodies were used to western blot. **Table S4.** Sequences of Primer for Real-time Polymerase Chain Reaction. **Figure S1.** High TPI1 expression in BRCA and biological analysis of co-expressed genes with TPI1. **Figure S2.** CDCA5 promotes breast cancer cell malignant phenotype.

## Data Availability

TCGA, UALCAN, METABRIC, cBioportal, GEPIA and Tang (2018) dataset of Kaplan–Meier plotter public databases were used in article. Other data supported the findings of this study could be available from the corresponding author on reasonable request.

## Abbreviations

TPI1: Triosephosphate isomerase 1
BRCA: Breast cancer
PI3K/AKT/mTOR: Phosphoinositide 3-kinase/AKT serine/threonine kinase/mammalian target of rapamycin
CDCA5: Cell division cycle associated 5
SQSTM1: Sequestosome-1
GAPDH: Glyceraldehyde-3-phosphate dehydrogenase
ICC: Intrahepatic cholangiocarcinoma
EMT: Epithelial–mesenchymal transformation
TCGA: The Cancer Genome Atlas Research Network
KM plotter: Kaplan–Meier plotter
OS: Overall survival
DFS: Disease-free survival
CHX: Cycloheximide
WB: Western blot
co-IP: Co-immunoprecipitation
IHC: Immunohistochemistry
IF: Immunofluorescence
ER: Estrogen receptor
PR: Progesterone receptor
GSEA: Gene set enrichment analysis
KEGG: Kyoto Encyclopedia of Genes and Genomes
