# Supplementary material for: TPI1 activates the PI3K/AKT/mTOR signaling pathway to induce breast cancer progression by stabilizing CDCA5
Source: J Transl Med. 2022 May 4;20:191. doi: 10.1186/s12967-022-03370-2 (PMC9066866; doi:10.1186/s12967-022-03370-2)
Supplement: Supplementary file 1 — Additional file 1: Table S1. Antibodies were used to IHC. Table S2. Sequences of the interference TPI1 are the followings. Table S3. Antibodies were used to western blot. Table S4. Sequences of Primer for Real-time Polymerase Chain Reaction. Figure S1. High TPI1 expression in BRCA and biological analysis of co-expressed genes with TPI1. Figure S2. CDCA5 promotes breast cancer cell malignant phenotype. [file 12967_2022_3370_MOESM1_ESM.docx]

**Table S1 Antibodies were used to IHC**

| **Antibodies** | | |
| --- | --- | --- |
| **REAGENT** | **Catalog and Source** | **Dilution** |
| TPI1 | Cat#10713-1-AP, Proteintech, | 1:200(human), 1:100(mouse) |
| CDCA5 | Cat#67418-1-Ig, Proteintech | 1:200(human), 1:50(mouse) |
| p-mTOR | Cat# AF3308; Affinity | 1:50(human), 1:30(mouse) |
| Ki67 | Cat#27309-1-AP; Proteintech | 1:100 (mouse) |
| E-cadherin | Cat#20874-1-AP; Proteintech | 1:100 (mouse) |
| LDHA | Cat#19987-1-AP; Proteintech | 1:100 (mouse) |

**Table S2** **Sequences of the interference TPI1 are the followings**

| **ShTPI1 1** |
| --- |
| Top strand: |
| 5’-GATCCGCCGTATCATTTATGGAGGCTCTGTGTTCAAGAGACACAGAGCCTCCATAAATGATACGGTTTTTTG-3’ |
| Bottom strand: |
| 5’-AATTCAAAAAACCGTATCATTTATGGAGGCTCTGTGTCTCTTGAACACAGAGCCTCCATAAATGATACGGCG-3’ |
| **ShTPI1 2** |
| Top strand: |
| 5’-GATCCGTCAAGCCCGAATTCGTGGACATCATTTCAAGAGAATGATGTCCACGAATTCGGGCTTGATTTTTTG-3’ |
| Bottom strand: |
| 5’-AATTCAAAAAATCAAGCCCGAATTCGTGGACATCATTCTCTTGAAATGATGTCCACGAATTCGGGCTTGACG |
| **ShTPI1 3** |
| Top strand: |
| 5’-GATCCGCCCGAATTCGTGGACATCATCAATGTTCAAGAGACATTGATGATGTCCACGAATTCGGGTTTTTTG-3’ |
| Bottom strand: |
| 5’-AATTCAAAAAACCCGAATTCGTGGACATCATCAATGTCTCTTGAACATTGATGATGTCCACGAATTCGGGCG-3’ |

**Table S3 Antibodies were used to western blot.**

| **Antibodies** | | |
| --- | --- | --- |
| **REAGENT** | **Catalog and Source** | **Dilution** |
| TPI1 | Cat#10713-1-AP, Proteintech, | 1:1000 |
| E-cadherin | Cat#3195; Cell Signaling Technology  Cat#WL01482; wanleibio | 1:1000  1:1000 |
| N-cadherin | Cat#13116; Cell Signaling Technology | 1:1000 |
| Vimentin | Cat#10366-1-AP; Proteintech | 1:1000 |
| t-PI3K | Cat# 11889, Cell Signaling Technology | 1:1000 |
| p-PI3K (Tyr199) | Cat#4228, Cell Signaling Technology | 1:800 |
| t-AKT | Cat#4685; Cell Signaling Technology | 1:1000 |
| p-AKT(Ser473) | Cat#9271; Cell Signaling Technology | 1:1000 |
| t-mTOR | Cat#2983; Cell Signaling Technology | 1:1000 |
| p-mTOR(Ser 2448) | Cat#5536; Cell Signaling Technology | 1:1000 |
| t-70s6k | Cat#34475; Cell Signaling Technology | 1:1000 |
| p-70s6k  (Thr421/Ser424) | Cat#9204; Cell Signaling Technology | 1:1000 |
| CDCA5 | Cat#67418-1-Ig, Proteintech  Cat#sc-365319, Santa Cruz | 1:800  1:500 |
| SQSTM1/p62 | Cat#66184-1-Ig, Proteintech | 1:1000 |
| LDHA | Cat#19987-1-AP; Proteintech | 1:1000 |
| PGK1 | Cat#17811-1-AP; Proteintech | 1:1000 |
| ENO1 | Cat#11204-1-AP; Proteintech | 1:1000 |

**Table S4 Sequences of Primer for Real-time Polymerase Chain Reaction**

| **TPI1** |  |
| --- | --- |
| Forward | 5’- CCCAGGAAGTACACGAGAAG-3’ |
| Reverse | 5’-CAGTCACAGAGCCTCCATAAA-3’ |
| **CDCA5** |  |
| Forward | 5’-CCCGAGAAACAGAAACGTAAGA-3’ |
| Reverse | 5’-TCATTCAACCACGGAGATCAAAC-3’ |
| **SQSTM1** |  |
| Forward | 5’-GGAACAGATGGAGTCGGATAAC-3’ |
| Reverse | 5’-ATCTGTAGGGACTGGAGTTCA-3’ |
| **GAPDH** |  |
| Forward | 5’-GGTATGACAACGAATTTGGC-3’ |
| Reverse | 5’-GAGCACAGGGTACTTTATTG-3’ |

**
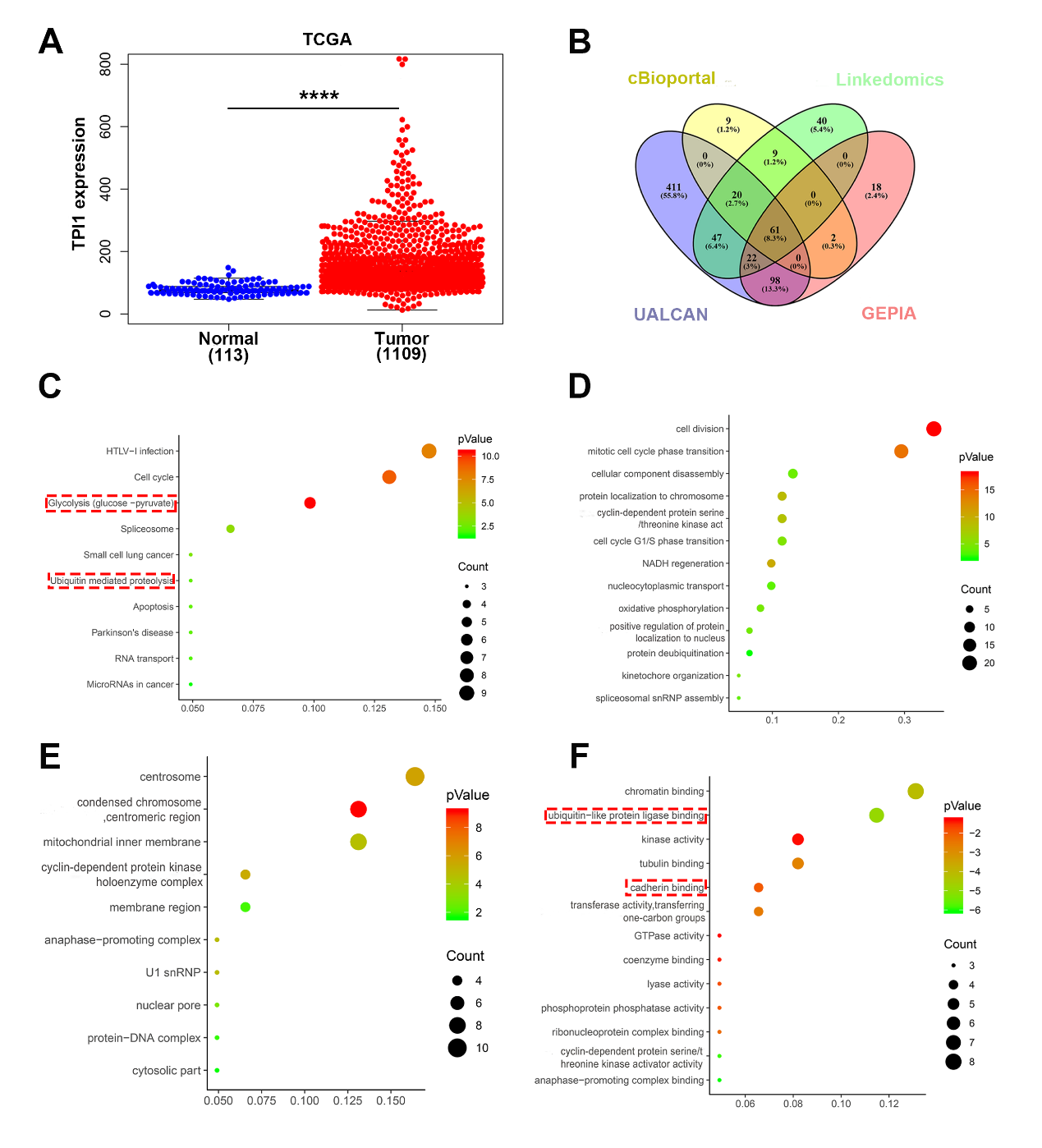
**

**Figure S1. High TPI1 expression in BRCA and biological analysis of co-expressed genes with TPI1.**

(**A**) Th expression of TPI1 was significantly higher in 1109 BRCA tissues than that in 113 normal tissues (TCGA). (*****p*<0.0001). (**B**) The Venn diagram (<https://bioinfogp.cnb.csic.es/tools/venny/index.html>) showed 61 genes that were significant associated with TPI1 in BRCA from the intersection of cBioportal database, Linkedomics database, GEPIA database and UALCAN database. (**C**) Co-expressed 61 genes were used for KEGG pathway enrichment analysis. (**D** - **F**) GO enrichment of co-expressed genes in biological process(D), cellular component and (E) and molecular function(F). (P represents the inverse log 10 of FDR values).

**
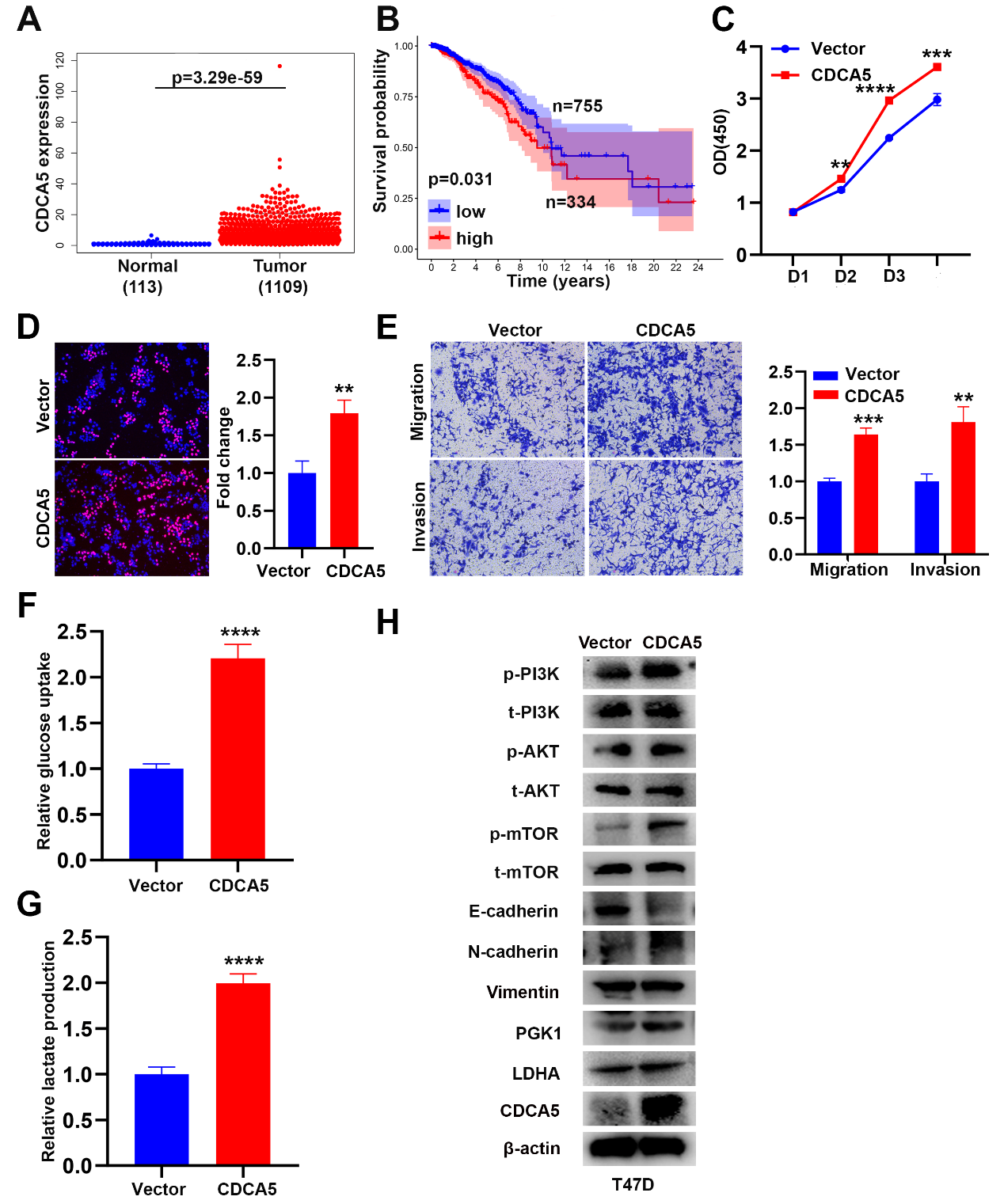
 Figure S2. CDCA5 promotes breast cancer cell malignant phenotype.**

(**A**) Expression of CDCA5 was significantly higher in BRCA tissues than that in 113 normal tissues using the Wilcoxon signed-rank test (TCGA). (**B**) Kaplan–Meier plotter showed the high expression of CDCA5 was associated with worse OS from TCGA database. (**C-D**) CCK-8 (C) and EDU(D) assays showed proliferation function of CDCA5. (**E**) Transwell assay shows migration and invasion in T47D cells. (**F-G**) Glucose uptake (F) and lactate production(G) were showed in T47D cells. (**H**) Western blot analysis key protein expression of PI3K/AKT/mTOR pathway, EMT markers and glycolysis markers in T47D cell after overexpression CDCA5 (***p*< 0.01, ****p* < 0.001, *****p*< 0.0001).
